# Characterization and Microstructure of Linear Electrode-Electrospun Graphene-Filled Polyvinyl Alcohol Nanofiber Films

**DOI:** 10.3390/ma11061033

**Published:** 2018-06-19

**Authors:** Ting-Ting Li, Mengxue Yan, Qian Jiang, Hao-Kai Peng, Jia-Horng Lin, Ching-Wen Lou

**Affiliations:** 1 Innovation Platform of Intelligent and Energy-Saving Textiles, Tianjin Polytechnic University, Tianjin 300387, China; litingting_85@163.com (T.-T.L.); 13032210827@163.com (M.Y.); jiangqian@tjpu.edu.cn (Q.J.); skyphk@163.com (H.-K.P.); jhlin@fcu.edu.tw (J.-H.L.); 2 Fujian Key Laboratory of Novel Functional Fibers and Materials, Minjiang University, Fuzhou 350108, China; 3 Laboratory of Fiber Application and Manufacturing, Department of Fiber and Composite Materials, Feng Chia University, Taichung 40724, Taiwan; 4 Department of Chemical Engineering and Materials, Ocean College, Minjiang University, Fuzhou 350108, China; 5 Department of Fashion Design, Asia University, Taichung 41354, Taiwan; 6 School of Chinese Medicine, China Medical University, Taichung 40402, Taiwan; 7 College of Textile and Clothing, Qingdao University, Shandong 266071, China; 8 Department of Bioinformatics and Medical Engineering, Asia University, Taichung 41354, Taiwan

**Keywords:** linear electrode-electrospun, nanofiber films, graphene (GR), polyvinyl alcohol (PVA), water contact angle, thermal stability, electromagnetic interference shielding (EMSE)

## Abstract

With the aim of achieving controllable mass production of electrospun nanofiber films, this study proposes and investigates the feasibility of using a custom-made linear electrode- electrospun device to produce conductive graphene (GR)-filled polyvinyl alcohol (PVA) nanofibers. The film morphology and diameter of nanofibers are observed and measured to examine the effects of viscosity and conductivity of the PVA/GR mixtures. Likewise, the influence of the content of graphene on the hydrophilicity, electrical conductivity, electromagnetic interference shielding effectiveness (EMSE), and thermal stability of the PVA/GR nanofiber films is investigated. The test results show that the PVA/GR mixture has greater viscosity and electric conductivity than pure PVA solution and can be electrospun into PVA/GR nanofiber films that have good morphology and diameter distribution. The diameter of the nanofibers is 100 nm and the yield is 2.24 g/h, suggesting that the process qualifies for use in large-scale production. Increasing the content of graphene yields finer nanofibers, a smaller surface contact angle, and higher hydrophilicity of the nanofiber films. The presence of graphene is proven to improve the thermal stability and strengthens the EMSE by 20 dB at 150–1500 MHz. Mass production is proven to be feasible by the test results showing that PVA/GR nanofiber films can be used in the medical hygiene field.

## 1. Introduction

Polyvinyl alcohol (PVA) is a highly hydrophilic, biocompatible, and biodegradable polymer [1,2,3] with good chemical stability and mass transfer properties [4,5,6]. PVA nanofibers can be used as wound dressings, drug carriers, biomedical materials, and matrices for tissue regeneration [7,8,9]. The drawback of PVA nanofiber films is with respect to their low mechanical properties. The addition of nanofillers can improve mechanical, electrical, thermal, and optical properties. For example, graphene (GR) is one commonly used nanofiller [10,11] capable of increasing the mechanical properties considerably and retaining the intrinsic biocompatibility, which massively strengthens the polymer matrix composites [12,13]. Graphene also features a high specific surface area, surface conductivity, and transmission capacity, and even accelerates the transmission of drugs and target cells [14,15,16,17,18,19].

Nanofibers have a tremendously high specific surface area and mass ratio, both of which are inversely proportional to the diameter, and can achieve a greater diameter ratio and porosity [20,21,22]. Hence, nanofibers are commonly seen in biomedical, environmental, and optoelectronic applications [23]. In addition to the micro/nano processing methods including photolithography, electron beam exposure, and ion beam cutting, nanofibers can also be produced through vapor deposition methods such as the template method, self-assembly solution growth method, nanoimprinting, and electrospun [24,25]. By contrast, the electrospun technique is a newer and more efficient, low-cost, non-polluting method that has been proven to be the most effective and direct technique. Electrospun nanofibers, which have been widely used due to their efficient properties [26], can be produced by needleless electrospun and needle electrospun.

Needleless electrospun avoids clogged needles and magnificently increases the spinning efficiency and the yields of nanofibers [27]. The process of needleless electrospun has undergone development. In addition to the magnetic fluid auxiliary electrospun [28] and bubble electrospun [29], other needleless electrospun methods use different spinnerets such as cylinders [30], conical coils [31], pyramids [32], disk nozzles, and spirals [33,34]. The drawback is that the jet flow is directly drawn from the free surface of the electrospun liquid to form nanofibers, and the unpredictable process difficult to manage [35]. Changing the spinning electrode is one measure to secure the spinning process to a certain extent. For example, Niu et al. invented a spinning electrode in a spiral line and obtained a more powerful and more even electric field surrounding the jet than that of cylinder and disk nozzles electrodes. The nanofibers were of better quality and could be produced in greater quantity [33,34]. The advanced study by Huang et al. produced GR-PVA nanofibers using the electrospun technique, and the microscopic structure of graphene nanosheets (GNS)/PVA nanofibers was observed. The average diameter of the nanofibers was 371 nm [7]. Golafshan et al. investigated the graphene/poly (vinyl alcohol)/sodium alginate (Gr-AP) fibrous scaffolds for engineering neural constructs and found that the scaffolds that were composed of 1 wt % Gr-AP had an average nanofiber diameter of 296 ± 40 nm [36].

Nevertheless, there are relatively fewer studies incorporating needleless electrospun with the preparation of PVA/GR nanofiber films. In this study, the custom-made copper wires are used as the spinning electrode for the electrospun of the PVA/GR nanofiber films with a finer diameter, and the nanofiber films are then evaluated in terms of the potential of mass production. The influences of viscosity and conductivity of the PVA/GR mixtures on the morphology and diameter as well as the influence of the content of graphene on the wettability, thermal stability, electric conduction, and electromagnetic shielding performance of the PVA/GR nanofiber films are evaluated.

## 2. Experiments

### 2.1. Materials

Polyvinyl alcohol (PVA, Changchun Chemical, Jiangsu, China) was purchased with a molecular weight of 84,000–89,000 g/mol. Sodium dodecyl sulfate (SDS) was purchased from Shanghai Macklin Biochemical Co. Ltd, Shanghai, China. Graphene (GR, P-ML20) was purchased from Enerage Inc., Yilan, Taiwan.

### 2.2. Preparation of PVA/GR Nanofiber Films

**Figure 1:**
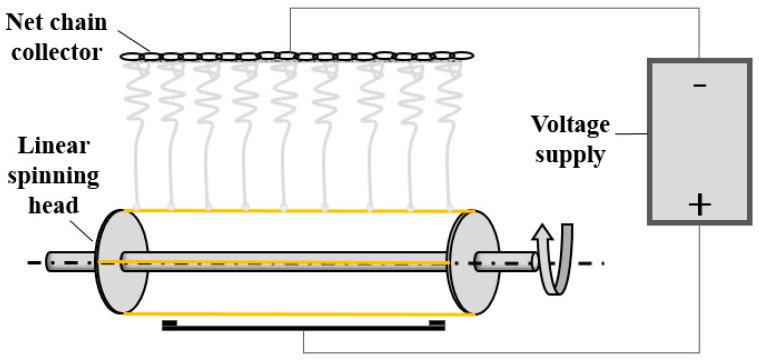
Schematic diagram of the copper linear electrode for electrospun.

Graphene (0, 0.01, 0.1, 0.25, 0.5, 1, and 2 wt %) was added to 1 wt % SDS with ultrasonic treatment for 3 h, after which PVA powders were added with magnetic stirring at 90 °C for 2 h and ultrasonic treatment for 3 h, forming different PVA/GR mixtures. Based on our previous study on the spinnability of PVA using a linear electrode, the PVA solution had a specified concentration of 7.5 wt % [37]. The electric conductivity and viscosity of the PVA/GR mixture were measured using a portable multiple parameter tester (ST3100MZH/F, OHAUS, Pine Brook, NJ, USA) and a digital viscosity meter (Bangxi Instrument Technology, Shanghai, China). Afterwards, the PVA/GR mixtures underwent needleless electrospun into nanofiber films at 25 °C with a humidity of 23% using a linear electrospun device which included a linear spinning head, a high-voltage power supply, and a grounded mesh collector (see Figure 1). The linear nozzle had a length of 15 cm and a diameter of 0.8 mm. The rate of the linear spinning head was 72 r/h, with a spinning voltage of 70 kV and a spinning distance of 30 cm [37]. The pure PVA nanofiber films represented the control group and the PVA/GR nanofiber films the experimental group. Both the control group and the experimental group were adhered with aluminum foil for electromagnetic interference shielding effectiveness (EMSE) measurement only.

### 2.3. Morphology and Characterizations of PVA/GR Nanofiber Film

Scanning electron microscopy (SEM, TM3030, HITACHI, Tokyo, Japan) was used to observe the morphology of the nanofibers. A bundle of 100 nanofibers was used to compute the average diameter. A surface contact angle instrument (JC2000DM, Shanghai Zhongchen Digital Technic Apparatus, Shanghai, China) and deionized water were used to measure the surface contact angle at 25 °C every 10 seconds, thereby examining the wettability of the PVA/GR nanofiber films. The thermogravimetric (TG) measurement was conducted using a thermogravimetric analyzer (TG 209F3, NETZSCH, Bavaria, Germany) with nitrogen gas at a flow rate of 60 mL/min. The relative mass loss of the samples was recorded from 25 °C to 700 °C with a heating rate of 10 °C/ min, thereby characterizing the thermal stability of the PVA/GR nanofiber films. A surface resistance instrument (RT-1000, OHM-STAT, Static Solutions Inc., Hudson, NY, USA) was used to measure the surface resistivity of the PVA/GR nanofiber films as specified in JIS L1094. The instrument equipped a 5-pound weight ensured that the two parallel electrodes were in good contact with the surface of the sample. Twenty samples for each specification were taken for the mean. The EMSE of PVA/GR nanofiber films shielding electromagnetic waves at frequencies between 0.1 MHz and 1.5 GHz was measured using an EMSE tester (EM-2107A, TS RF Instrument, Taoyuan, Taiwan) as specified in ASTM D4935. The cylinder samples had a diameter of 80 mm.

## 3. Results and Discussion

### 3.1. Properties of GR/PVA Mixtures

Different amounts of graphene were added to 7.5 wt % PVA solution, and the mixtures were made into nanofibers using needleless electrospun. Table 1 shows the viscosity of different PVA/GR mixtures at 25 °C. The viscosity of the pure PVA solution is 254 mPa∙s., while the PVA/GR mixtures have a viscosity that first increases and then declines when the constituent graphene is over 0.25 wt %. The highest and lowest viscosity values of 485 mPa∙s. and 155.5 mPa∙s were obtained when the PVA/GR mixtures consisted of 0.25 wt % and 1 wt % graphene, respectively. Viscosity is dependent on the molecular weight and molecular chain entanglement. Excessive graphene liberates the entanglement of PVA molecular chains, which causes a drastic decrease in the degree of entanglement [7,38,39,40]. Table 1 shows the electric conductivity of PVA/GR mixtures and that of pure PVA solution of 0.67 mS/cm. Graphene is the conductive filler, and the greater the quantity of graphene, the greater the electric conductivity of the PVA/GR mixture. The PVA/GR mixture containing 1 wt % graphene has an optimal conductivity of 2.03 mS/cm, which is 1.36 mS/cm higher than that of the pure PVA solution. The increment in conductivity then becomes mild due to the fact that excessive graphene easily agglomerates and precipitates [7,40].

### 3.2. Morphology and Diameter of PVA/GR Nanofiber Films

**Figure 2:**
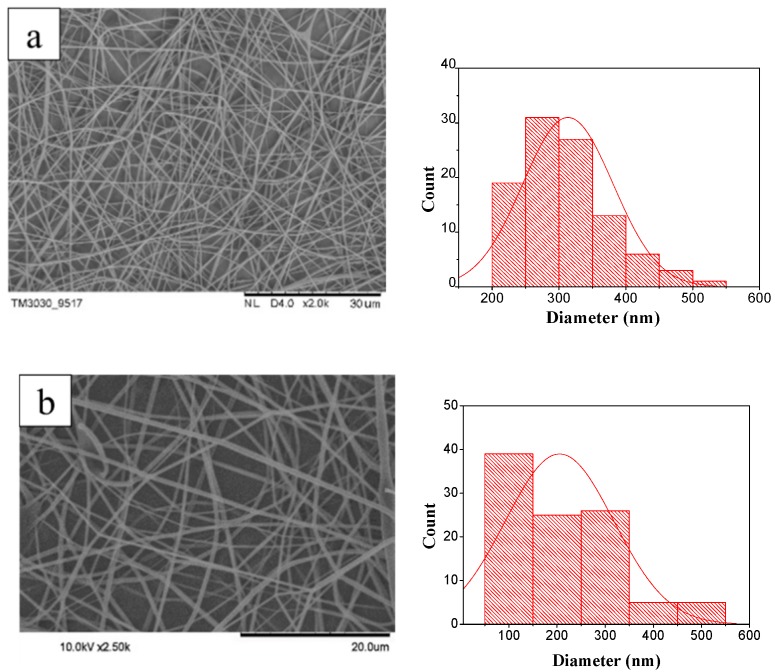

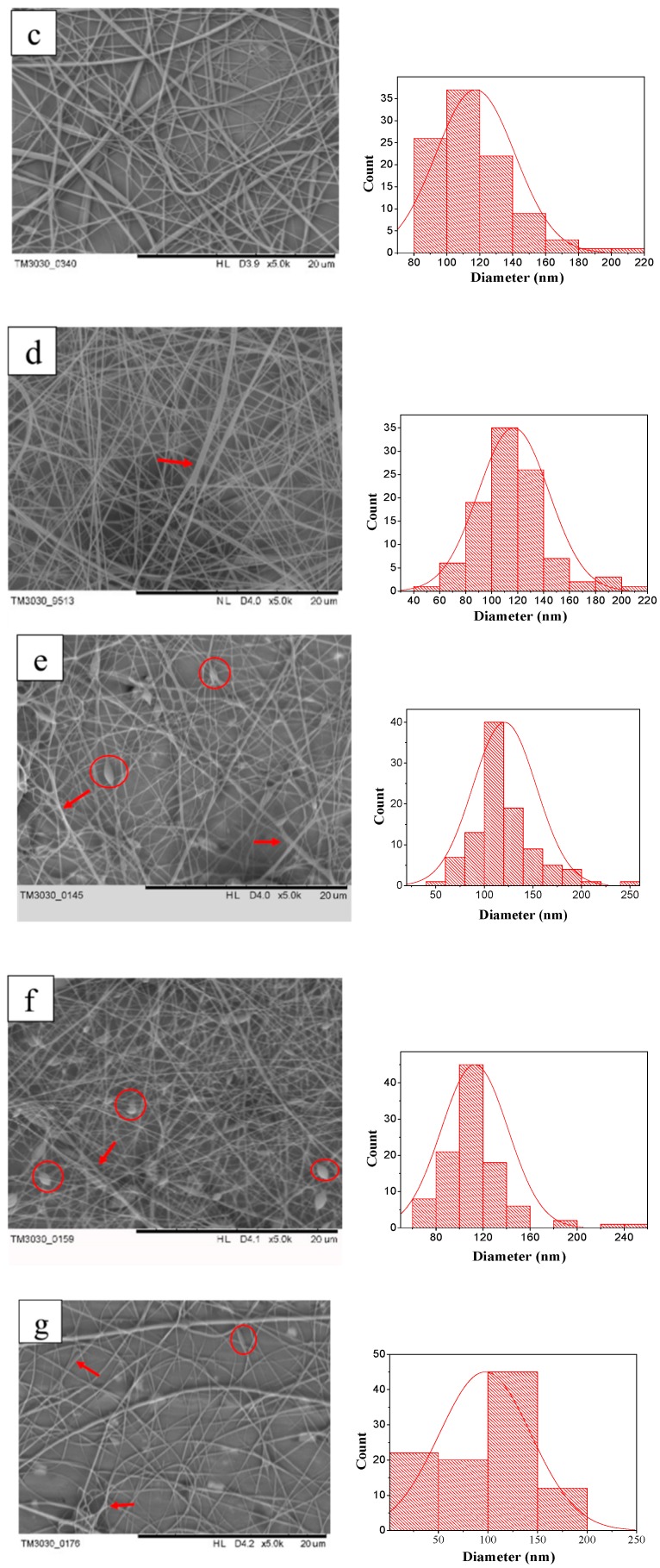
SEM images and corresponding diameter distribution of Polyvinyl alcohol/graphene (PVA/GR) nanofiber films made of 7.5 wt % PVA solution and (**a**) 0, (**b**) 0.01, (**c**) 0.1, (**d**) 0.25, (**e**) 0.5, (**f**) 1, and (**g**) 2 wt % graphene. The coalesced nanofibers and bead-shaped nanofibers are indicated in red arrows and by red circles, respectively. GR: graphene.

Figure 2 shows the SEM images and diameter distribution of the PVA/GR nanofiber films. The morphology and diameter distribution of the nanofibers are observed and measured in order to examine the influence of the content of graphene. The SEM images in Figure 2a–g show that increasing graphene is also detrimental to the spinnability and evenness of the nanofibers in addition to providing a rough surface. The adhesion between fibers is enhanced with more graphene, and some nanofibers are even coalesced. A content of graphene of 0.5 wt % leads to the presence of bead-shaped fibers (Figure 2f). Moreover, the number of bead-shaped nanofibers increases when the PVA/GR mixture is composed of 1 wt % graphene (Figure 2g).

**Figure 3:**
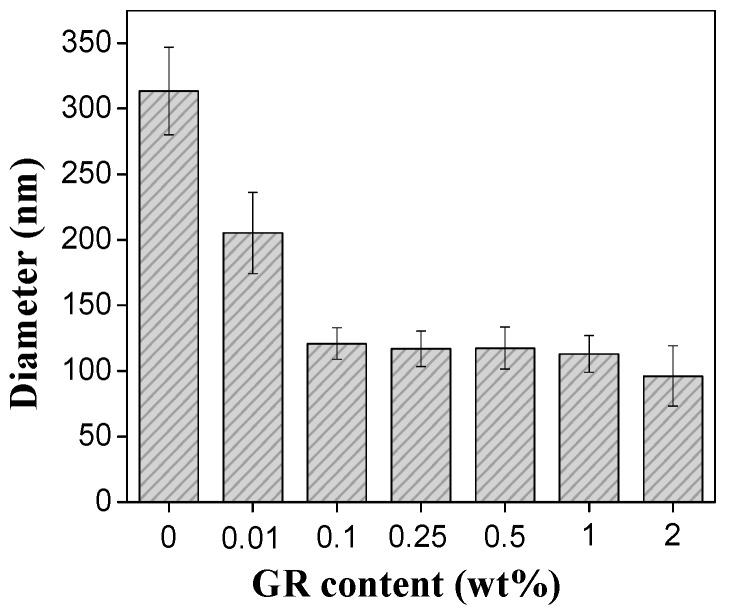
Diameter distribution of PVA/GR nanofiber films as related to the contents of graphene.

Figure 3 shows the diameter distribution of PVA/GR nanofibers. The PVA/GR mixtures are made of 7.5 wt % of PVA solution and 0.01, 0.1, 0.25, 0.5, or 1 wt % graphene. A greater proportion of graphene decreases the diameter of the nanofibers. In particular, when graphene is of 0.1 wt %, the diameter is 120 nm, which is 61% lower than that of pure PVA nanofibers. It is also 67% lower than the diameter (371 nm) of the graphene nanosheets (GNS) /PVA nanofibers proposed by Huang et al. [7]. Graphene is a highly conductive filler, and greater quantities of graphene have a positive influence on the static electricity which strengthens the traction onto the droplets and thus results in finer nanofibers [38,39]. Although the viscosity of PVA/GR mixture increases as a result of the increasing graphene, the viscosity has less influence than the electric conductivity of the mixture. As a result, the nanofibers have a greater fineness when the PVA/GR mixture consists of more graphene [7].

### 3.3. Dynamic Hydrophilicity of PVA/GR Nanofiber Films

**Figure 4:**
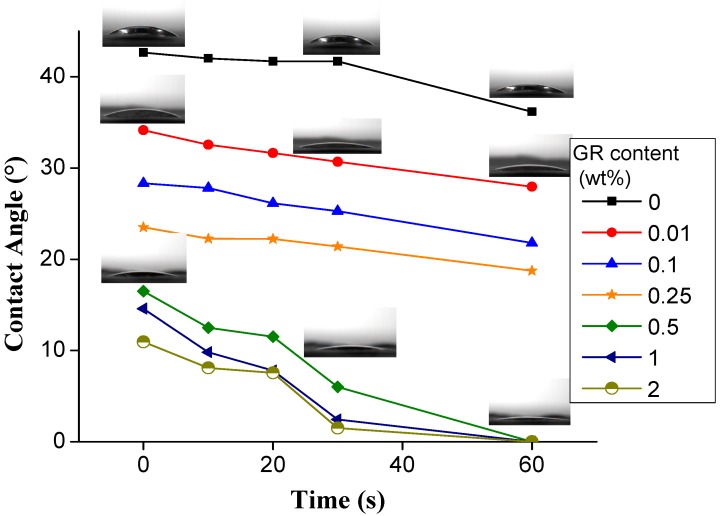
Dynamic hydrophilicity angle of PVA/GR nanofiber films as related to the content of graphene.

Figure 4 shows that the surface contact angle of pure PVA nanofiber films is 42.66°, which then gradually decreases with the increasing standing time. PVA has considerable hydrophilic -OH groups, which results in a high affinity with water molecules and provides nanofibers with greater moisture absorption capacity [41,42]. The surface contact angle diminishes from 42° to 10° as a result of increasing graphene. This decreasing trend conforms well to that related to graphene oxide content as proposed by Yang et al. and Da et al. [43,44]. Namely, when composed of more graphene, the nanofiber films have greater wettability. Based on Figure 3, the diameter of nanofibers is inversely proportional to the content of graphene. The finer nanofibers mean that the nanofiber films have a high porosity, and thus greater wettability. In addition, graphene is distributed in the interior and surface of the fiber film, and the addition of graphene increases the distance between the fibers, which facilitates the penetration of water into the membrane [43]. Moreover, the addition of graphene also renders the nanofibers with a rough surface. According to the Wenzel Equation (1), the relationship between the wetting angle of a rough interface (i.e., θ*) and a wetting angle (i.e., θ) can be presented as in Equation (1). When r > 1 and the angle is smaller than 90°, the nanofibers have a rougher surface and a smaller surface contact angle [41,42].

(1) $${\cos\theta }^{*}=\frac{f\left(\gamma_{CL}+\gamma_{CS}\right)}{\gamma_{SL}}=f\cos\theta$$

### 3.4. Thermal Stability of PVA/GR Nanofiber Films

**Figure 5:**
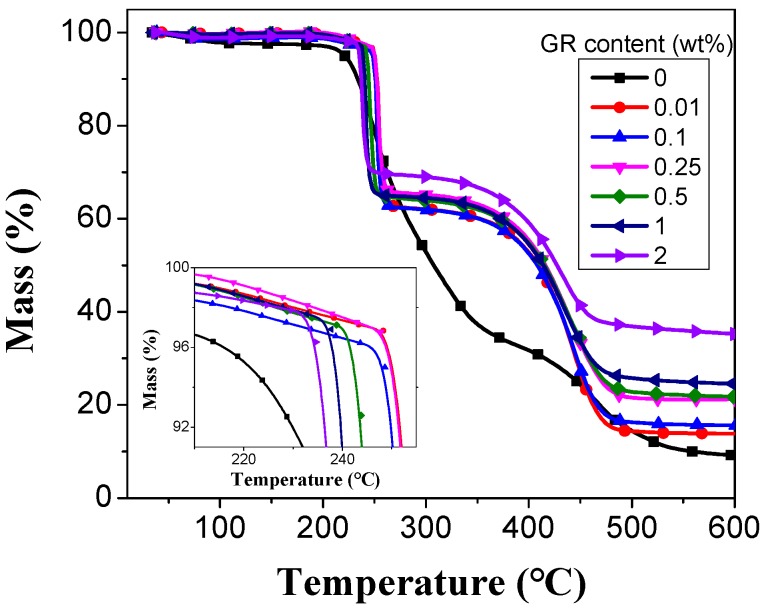
Thermogravimetric (TG) curves of PVA/GR nanofiber films as related to the content of graphene.

Figure 5 demonstrates the three stages of the TG curves of pure PVA and PVA/GR nanofiber films. The first stage shows the evaporation of water that PVA films and PVA/GR nanofiber films absorb before reacing200 °C. The second stage is a period of drop mass loss. The weight loss of PVA and PVA/GR nanofiber films at 200–400 °C is related to the decomposition of polar groups as well as to the dehydration of polymers and the formation of polyacetylene structures. In the third stage, main molecule chains of PVA degrade, releasing CO_2_ and forming oxides at a temperature between 400 °C and 550 °C [45,46]. Table 2 shows that at 700 °C, the pure PVA nanofiber films have 9.36% residue mass, and the PVA/GR nanofiber films containing 1 wt % graphene have 23.57% residue mass, which is 14% lower than that of the former. At 350 °C, the mass of pure PVA is 38.0%, but after addition of graphene it significantly increases to over 60%. This indicates that graphene addition improves the decomposition temperature of the PVA polar group. Moreover, the maximum decomposition temperature is raised between 0.01–0.25 wt % graphene additions. It is thus clear that PVA/GR nanofiber films have higher thermal stability at the second and third stages when composed of more graphene, which indicates the presence of graphene clearly improves the thermal stability of PVA/GR nanofiber films. PVA/GR nanofiber films degrade at 200–400 °C at the second stage due to decomposition of polar group and 400–550 °C at the third stage due to degradation of the main PVA chains. However, PVA/GR nanofiber films made of 0.5 wt % of graphene have a lower thermal stability because an excessive amount of graphene is somehow detrimental to the entanglement of molecules.

### 3.5. Electrical Property of PVA/GR Nanofiber Films

**Figure 6:**
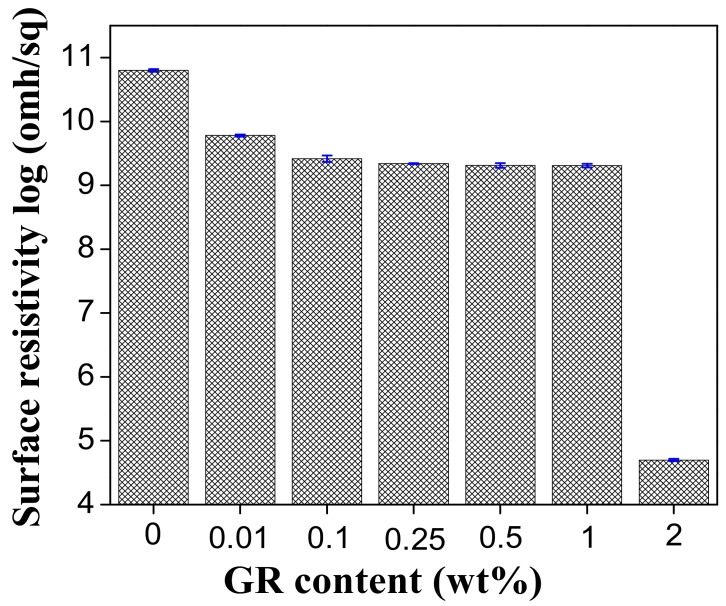
Surface resistivity of PVA/GR nanofiber films as related to the content of graphene.

Figure 6 shows the surface resistivity of the PVA/GR nanofiber films as related to the contents of graphene. The surface resistivity gradually decreases when the PVA/GR nanofiber films consist of more graphene. Graphene, a conductive filler, strengthens the electric conductivity of the films. The conductive network is better constructed as a result of increasing the conductive graphene [36]. The surface electric resistivity of the pure PVA nanofiber films is 6.26 × 10^10^ ohms/sq, and that of the PVA/GR nanofiber films with 2 wt % of graphene is 4.99 × 10^4^ ohms/sq.

**Figure 7:**
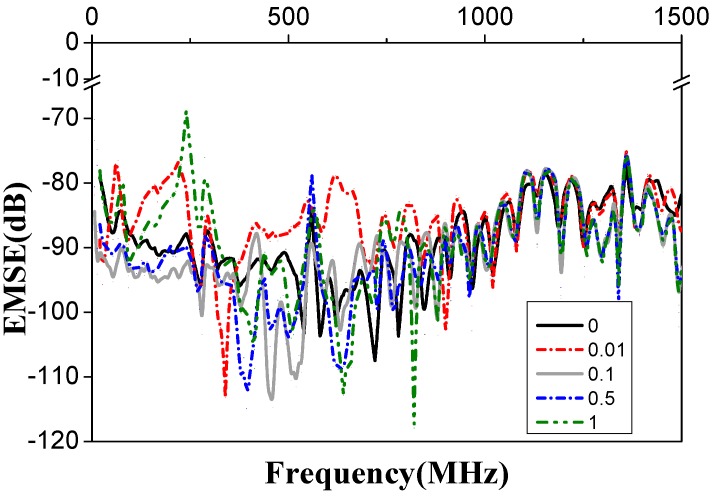
Electromagnetic interference shielding effectiveness (EMSE) curves of the PVA/GR nanofiber films as related to the content of graphene (0.01, 0.1, 0.5, and 1 wt %). Both the experimental and control groups consist of an aluminum foil.

Figure 7 shows the EMSE of the PVA/GR nanofiber films adhered with an aluminum foil (i.e., the experimental group) and the EMSE of the pure PVA nanofiber films attached with an aluminum foil (i.e., the control group). At frequencies of 250–1500 MHz, the experimental group has higher EMSE than the control group. When the frequency of electromagnetic waves is at 460 MHz, the EMSE of the control group is 93 dB and that of the experimental group containing 0.1 wt % graphene is 114 dB, a value 21 dB greater than for the control group. The electromagnetic waves are attenuated by reflection, absorption, and multiple absorption, and multiple reflection. The physical characteristics, organizational structure, and shape of the shielding materials are associated with the electromagnetic shielding effectiveness [44,47,48]. Aluminum foils use the reflection mechanism to debilitate the energy of electromagnetic waves, and the adhered PVA/GR nanofiber films have a porous structure, which is able to debilitate the incident waves via the multi-reflection [49]. In addition, the PVA/GR nanofiber films possess conductivity, and the electrically conductive composites can undermine electromagnetic waves via energy dissipation [50].

## 4. Conclusions

A custom-made linear electrode-electrospun technique is used to accomplish the controllable large-scale preparation of electrospun nanofiber membranes. The viscosity and conductivity of the mixture as well as the diameter and properties of the PVA/GR nanofiber films are evaluated in order to examine the influence of the content of graphene. The viscosity of the PVA/GR mixtures first decreases and then increases when the content of graphene increases, but the opposite is the case for the conductivity of the PVA/GR mixtures. The average diameter of the nanofibers is dependent on the conductivity of the mixture, and a small amount of graphene improves the fineness of the PVA/GR nanofibers. By contrast, an excessive amount of graphene causes bead-shaped and merged nanofibers. Specifically, 0.1 wt % graphene generates the optimal PVA/GR nanofiber films with 120-nm-thick nanofibers. Moreover, the addition of graphene has a negative influence on the surface contact angle. The PVA/GR nanofiber films containing 0.1 wt % graphene have a surface contact angle of 31°, which is 12° lower than that of the pure PVA nanofiber films. It is also equivalent to a greater wettability as a full saturation takes 190 s. The presence of graphene strengthens the thermal stability of PVA/GR nanofiber films at second and third stages, and the decomposition temperature reaches 255.2 °C. However, excessive graphene adversely affects the decomposition temperature. Compared to the pure PVA nanofiber films with a surface resistivity of 6.26 × 10^10^ (ohms/sq), the PVA/GR nanofiber films have a surface resistivity of 4.99 × 10^4^ (ohms/sq) due to the presence of graphene. Specifically, PVA/GR nanofiber films containing 0.1 wt % graphene have a surface resistivity of 2.60 × 10^9^ (ohms/sq), a maximum yield of 2.24 g/h, and an EMSE of 114 dB which is 21 dB greater than that of the control group. The test results serve a useful reference for the mass production of PVA/GR nanofiber films in the future that can be applied to the medical hygiene field.

## Figures and Tables

**Table 1:** Viscosity and conductivity of the polyvinyl alcohol (PVA) solution.

	Content of Graphene (wt %)						
	0	0.01	0.1	0.25	0.5	1	2
Viscosity (mPa∙s)	254.0	330.0	427.0	485.0	287.0	155.5	133.5
Electric conductivity (mS/cm)	0.67	1.67	1.99	1.93	1.97	2.03	1.98

**Table 2:** TG results of PVA/graphene (GR) nanofiber films.

GR Content (wt %)	0	0.01	0.1	0.25	0.5	1	2
Maximum decomposition temp. (°C)	248.9	255.2	253.2	254.7	247.7	242.9	238.6
Residual mass at 700 °C (%)	9.36	12.4	15.26	21.4	20.57	23.57	33.64
Mass at 350 °C (%)	38.0	60.1	60.4	63.1	62.0	62.6	66.8
